# Genome-wide association study of 17 serum biochemical indicators in a chicken F_2_ resource population

**DOI:** 10.1186/s12864-023-09206-7

**Published:** 2023-03-02

**Authors:** Haijie Song, Wenting Li, Yuanfang Li, Bin Zhai, Yujie Guo, Yi Chen, Ruili Han, Guirong Sun, Ruirui Jiang, Zhuanjian Li, Fengbin Yan, Guoxi Li, Xiaojun Liu, Yanhua Zhang, Yadong Tian, Xiangtao Kang

**Affiliations:** 1 College of Animal Science and Technology, Henan Agricultural University, No.15 Longzihu University Area, Zhengzhou New District, 450002 Zhengzhou, China; 2 Henan Key Laboratory for Innovation and Utilization of Chicken Germplasm Resources, Henan Agricultural University, No.15 Longzihu University Area, Zhengzhou New District, 450002 Zhengzhou, China

**Keywords:** Chicken, Genome-wide association study, 17 serum biochemical indicators

## Abstract

**Background:**

Serum biochemical indicators are often regarded as direct reflections of animal metabolism and health. The molecular mechanisms underlying serum biochemical indicators metabolism of chicken (Gallus Gallus) have not been elucidated. Herein, we performed a genome-wide association study (GWAS) to identify the variation associated with serum biochemical indicators. The aim of this research was to broaden the understanding of the serum biochemical indicators in chickens.

**Results:**

A GWAS of serum biochemical indicators was carried out on 734 samples from an F2 Gushi× Anka chicken population. All chickens were genotyped by sequencing, 734 chickens and 321,314 variants were obtained after quality control. Based on these variants, a total of 236 single-nucleotide polymorphisms (SNPs) on 9 chicken chromosomes (GGAs) were identified to be significantly (-log_10_(*P*) > 5.72) associated with eight of seventeen serum biochemical indicators. Ten novel quantitative trait locis (QTLs) were identified for the 8 serum biochemical indicator traits of the F2 population. Literature mining revealed that the *ALPL*, *BCHE*, *GGT2*/*GGT5* genes at loci GGA24, GGA9 and GGA15 might affect the alkaline phosphatase (AKP), cholinesterase (CHE) and γ-glutamyl transpeptidase (GGT) traits, respectively.

**Conclusion:**

The findings of the present study may contribute to a better understanding of the molecular mechanisms of chicken serum biochemical indicator regulation and provide a theoretical basis for chicken breeding programs.

**Supplementary Information:**

The online version contains supplementary material available at 10.1186/s12864-023-09206-7.

## Background

Conventional breeding methods are based mainly on phenotypic selection and have certain limitations. Fat composition, immunity and some other traits are difficult to measure directly and can be measured only in siblings or by slaughter, resulting in problems such as long generation intervals and high costs. In the chicken breeding industry, development of convenient measurement indicators that reflect physiological fat metabolism, immune function and other parameters is necessary. Serum biochemical indicators are considered reflections of animal metabolism and health status and have great significance in livestock and poultry production research. These indicators are also easy to measure. Analyses of serum biochemical indicators are helpful for indirect selection of some traits, assessment of animal health, early diagnosis of animal diseases and other procedures and effectively compensate for the shortcomings of long-term and high-cost conventional breeding. In addition, molecular breeding can accelerate the speed of animal breeding. Therefore, screening molecular markers and analyzing the genetic basis of serum biochemical indicators through GWAS is a promising avenue for exploiting genomic information for commercial breeding of chickens.

Serum enzymes are responsible for catalyzing various physiological and biochemical processes in animals, and their levels and activities reflect growth, development and metabolism levels to a certain extent. Alkaline phosphatase (AKP) is a metalloenzyme superfamily member that hydrolyzes or transphosphorylates various biomolecules and exhibits its highest biological activity in suitable alkaline environments [1]. AKP is widespread in nature and exists in almost all animal tissues. The levels of AKP are higher in the skeleton, liver and bile duct than in other tissues. AKP in serum mainly originates in the skeleton and liver. AKP levels are often used to diagnose and monitor skeletal or liver diseases in the clinic [2]. Valgus-varus deformity (VVD) is a leg disease which lateral or middle deviation of the tibiotarsus or tarsometatarsus, accompanied by abnormal changes in serum AKP levels. Case-control (VVD broilers-sound broilers)-based GWAS identified 43 SNPs located on Chr24 (0.22 Mb − 1.79 Mb) that were significantly associated with AKP [3]. In the chicken F2 resource population, the direct relationships between AKP levels and candidate genes and corresponding QTLs have not been reported at present. Cholinesterase (CHE) is an esterase that can break down choline esters and act as a neurotransmitter. Kidney disease, obesity, fatty liver, hyperthyroidism, and other conditions can lead to increased CHE levels. Measurement of serum CHE activity [4], serum alanine transaminase (ALT) levels and serum aspartate aminotransferase (AST) levels [5] is important for diagnosis of liver diseases, and ALT is a sensitive biochemical marker used to evaluate damage to hepatic parenchymal cells [5].

Blood lipid levels reflect overall lipid metabolism and health status. Abnormal blood lipid levels are associated with cardiovascular disease, familial hypercholesterolemia and diabetes. Clinical tests for total cholesterol (CHO), low-density lipoprotein cholesterol (LDL), high density lipoprotein cholesterol (HDL) and triglycerides (TGs) are widely used for cardiovascular disease risk assessment [6, 7]. Another important role of serum biochemical indicators is use them as indirect indicators for certain economic traits in animal breeding, such as the use of blood LDL as selection indicators for reducing abdominal fat content in broilers [8]. Fatty pigs have higher serum total protein (TP) levels than lean pigs, and serum TP level has been shown to be the best marker for early fat assessment in pigs [9, 10]. Multiple serum biochemical indicators have been identified as biomarkers for predicting important economic traits or general health in animals and humans, and deciphering the genetic basis of these biomarkers using GWAS is important for potential applications in animal breeding. Several genes associated with the electrolyte levels in serum were identified by case-control (health-disease) -based GWAS [11, 12]. Several early studies have reported associated QTLs for blood components in chickens [13–15]. As far as we know, no published study has systematically demonstrated the associations between the 17 serum biochemical indicators and genomic loci using a GWAS of SNPs in chickens.

Biochemical indicators in serum are direct reflections of metabolism and health in animals. Notably, different breeds of chickens have different growth and metabolism characteristics due to their different genetic backgrounds and thus show different levels of serum biochemical indicators. In the current study, we performed a GWAS of seventeen serum biochemical indicators in a total of 734 F_2_ chickens from Gushi chicken × Anka chicken populations to identify markers and candidate genes that affect serum biochemical indicators, laying a foundation to exploiting genomic information for commercial breeding of chickens.

## Results

### Phenotypes and phenotypic correlations

**Fig. 1:**
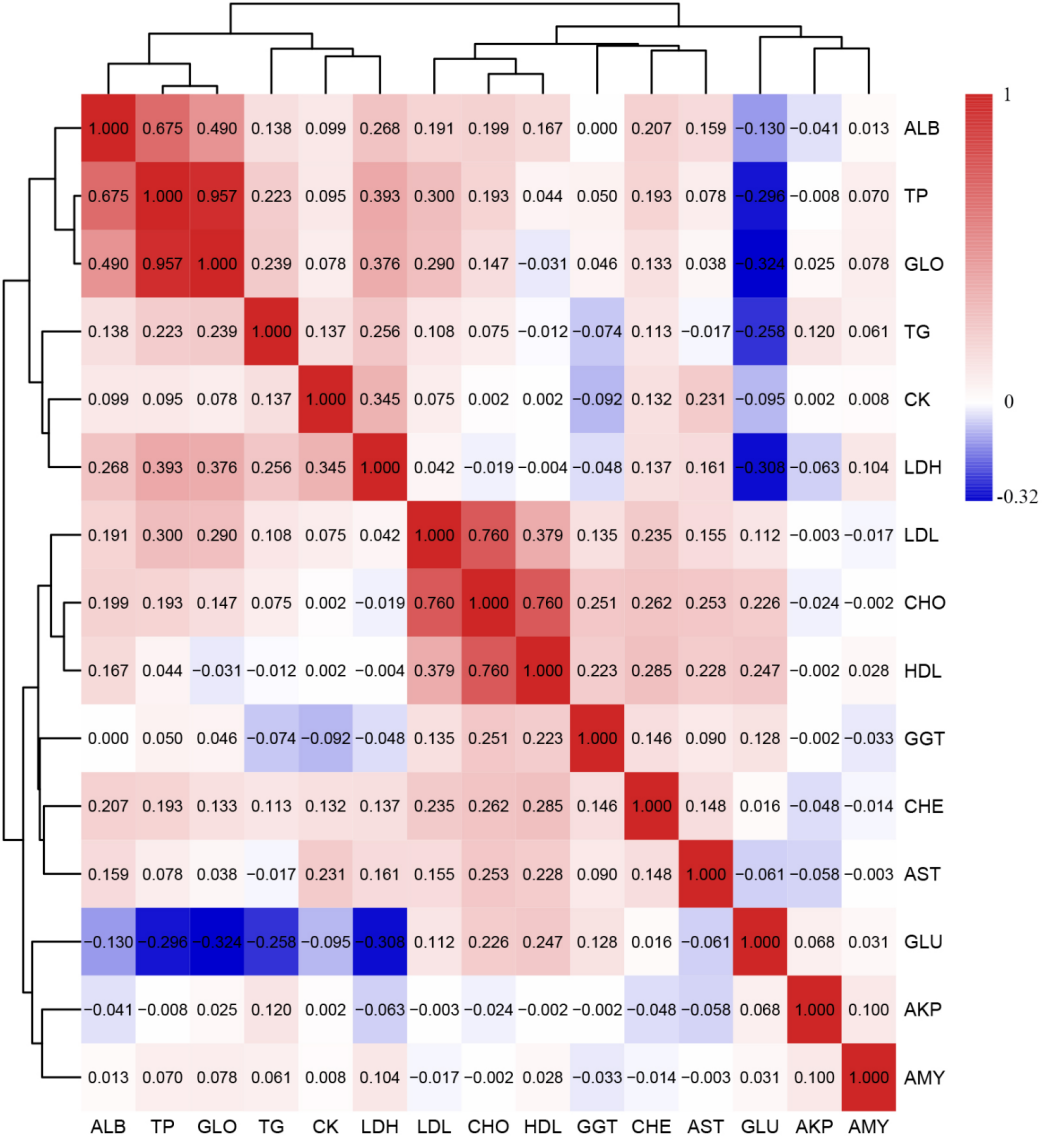
Pearson correlations between phenotypes. The Pearson correlation coefficients of pairs of traits were calculated, and the traits were clustered based on the correlation coefficients. The colors (numbers) represent the pairwise correlation coefficients of the serum biochemical indicators. Red indicates a positive correlation, and blue indicates a negative correlation

The descriptive statistics for the serum biochemical indicators are listed in Table S1. The variation was large for all serum biochemical indicators in the F_2_ resource population. The maximum coefficient of variation was 129.93% (for creatinine (CREA)), and the minimum was 13.17% (for albumin (ALB)). The pairwise correlation coefficients for the serum biochemical indicators (Table S2) were calculated and clustered (Fig. 1), and the results showed that ALB, TP, and globulin (GLO) were clustered together and strongly positively correlated, as were LDL, CHO, and HDL. In contrast, traits such as LDH, TP, GLO and TGs were negatively correlated with GLU.

### Summary of genotyping results

**Fig. 2:**
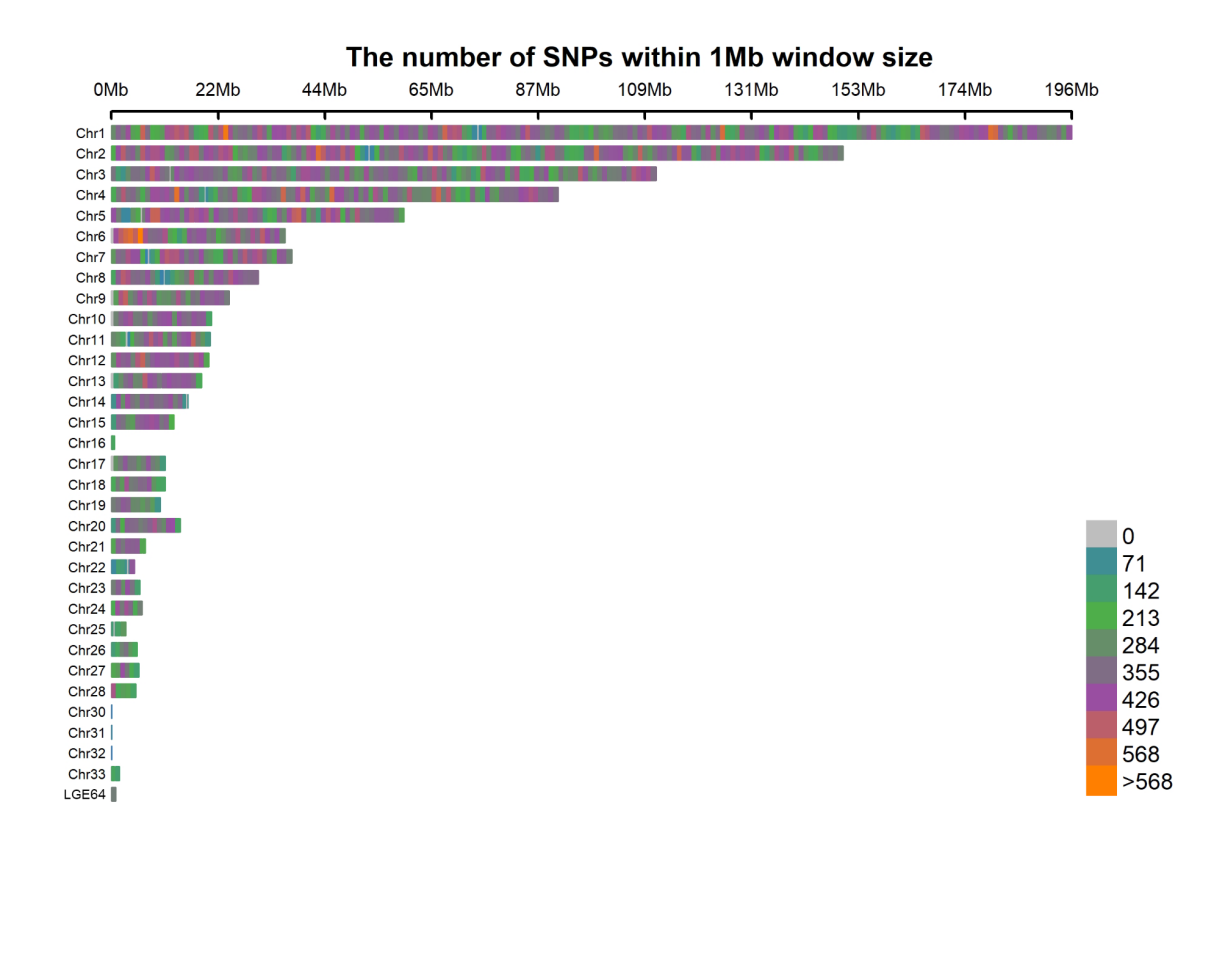
Density of SNPs in the chicken genome

**Fig. 3:**
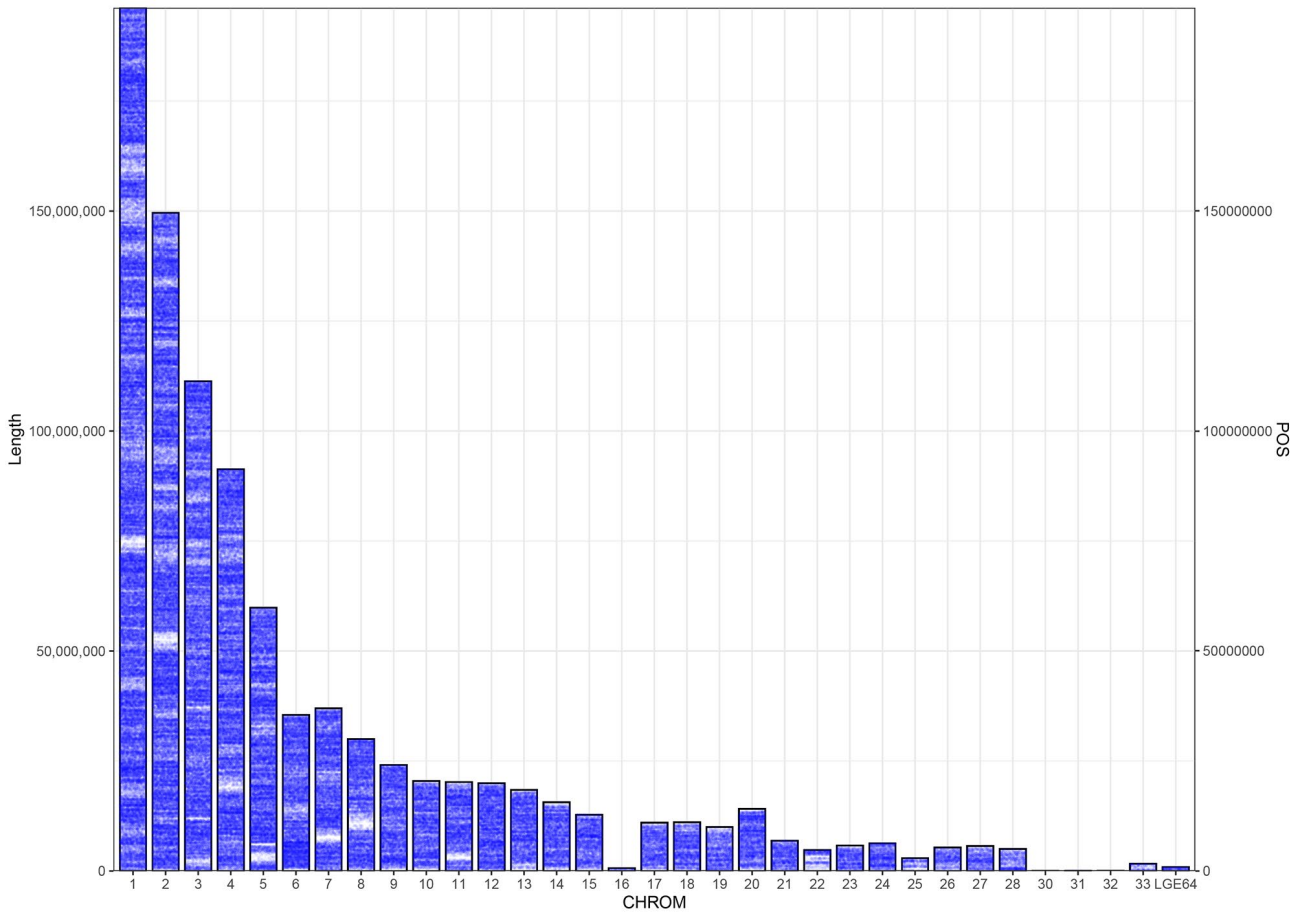
Distribution of SNPs in the chicken genome

**Fig. 4:**
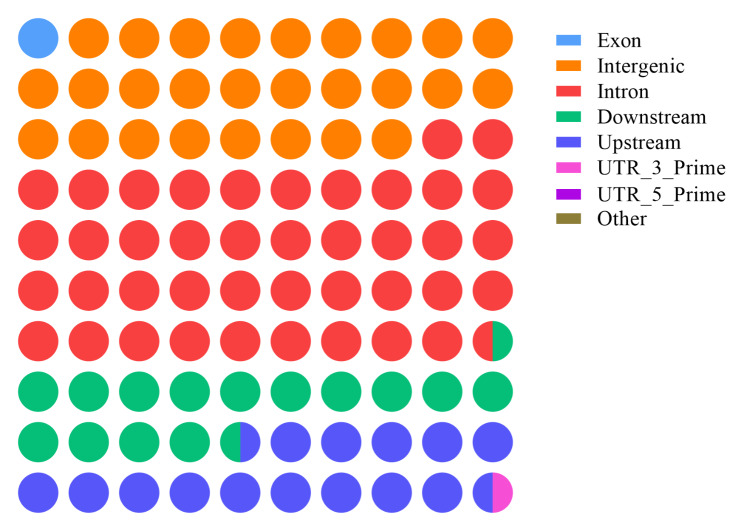
Annotated results of SNPs from GBS sequencing. A circle represents 1% of SNPs, and the color of the circle represents SNPs annotated to various functional regions

A total of 768 samples were genotyped by sequencing, which yielded 7.258 billion clean sequencing reads and 6.071 billion good barcode reads. The data quality parameters (Q20 and Q30) satisfied the requirements for the subsequent analyses (Table S3)^[16]^. After a series of quality control steps and removal of sex chromosomes, 734 chickens and 321,314 SNPs remained. Among these SNPs, ~ 10.59% (35,677 SNPs) were first identified by BLAST analysis of the NCBI chicken SNA database (dbSNP) (Table S4)^[16]^. The average SNP density and average SNP variant rate per chromosome were 309 SNPs/Mb and 5.79 kb/SNP, respectively (Fig. 2). These SNPs were evenly distributed on the genome (Fig. 3), and their distribution was not significantly related to GC content and repeat sequence distribution on the genome. Annotation analysis of the obtained SNPs, majority of the SNPs identified were located in the intergenic region (26.89%) and intron region (41.66%), and the proportion of SNPs in the exon region was 1.09% (Table S5 and Fig. 4).

### GWAS results for the 17 serum biochemical indicators

**Fig. 5:**
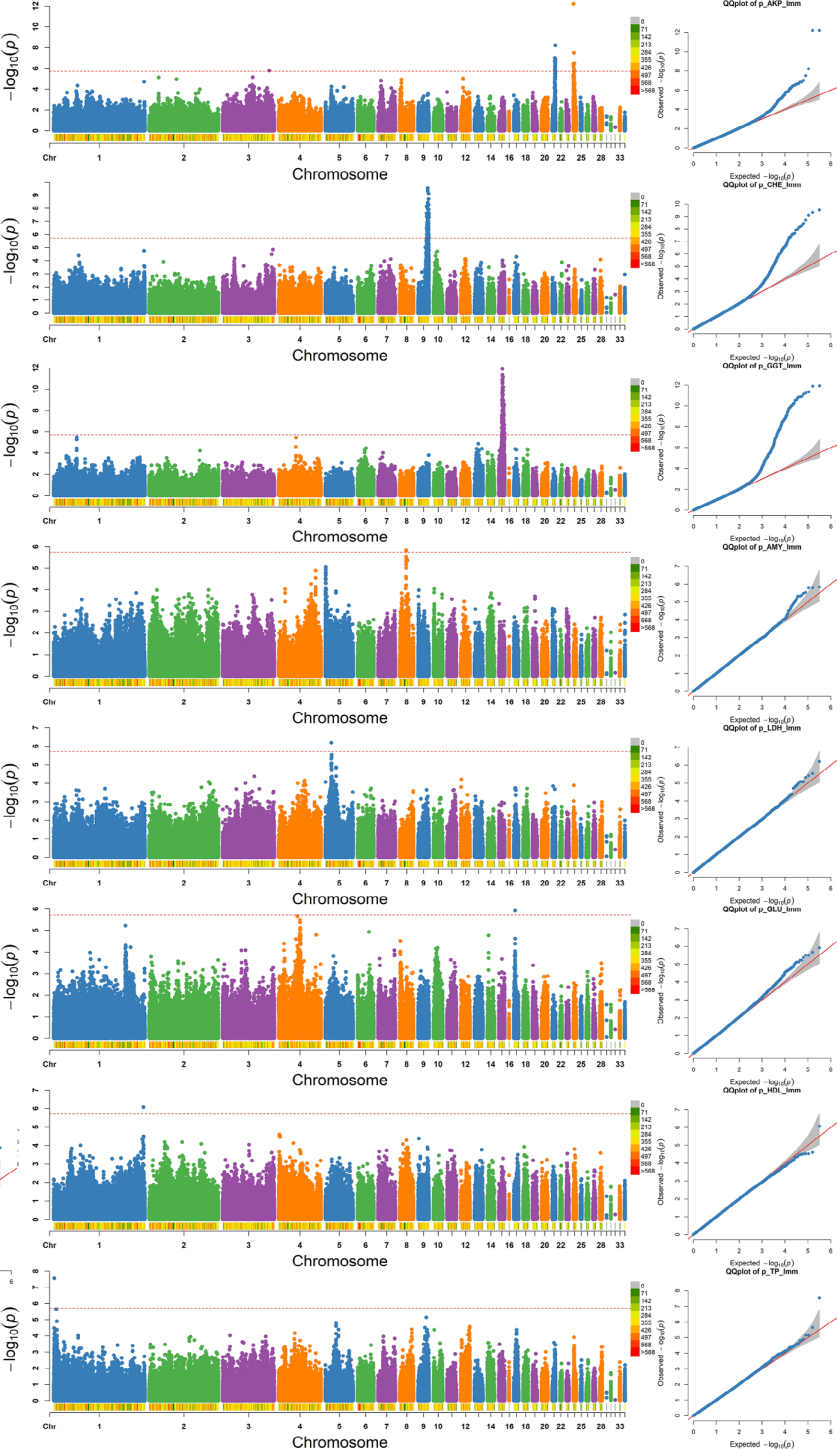
Manhattan plots (left) and Q-Q plots (right) for the 8 traits with significantly associated SNPs. Each dot in this figure corresponds to a SNP within the dataset. In each Manhattan plot, the dot color indicates the chromosome on which the SNP is located, the dot position indicates the -log_10_-transformed *P* value of the SNP, the number below represents the chromosome number, the length of the figure above the number represents the length of the chromosome, and the color represents the number of SNPs on the chromosome. The horizontal red dashed line denotes the genome-wide significance (-log_10_(*P*) = 5.72). For each Q-Q plot, the x-axis represents the expected -log_10_-transformed *P* value, the y-axis shows the observed -log_10_-transformed *P* value, and the red line is the diagonal line

A GWAS was carried out on 17 serum biochemical indicators in 734 individuals from the Gushi×Anka F_2_ population with an MLM using GCTA. The results of the GWAS are shown in Fig. 5 and Figure S1. The single-marker analysis identified 236 SNPs significantly associated with 8 of the 17 traits with genome-wide significance (-log_10_(P) > 5.72), which were mapped to GGA1, GGA3, GGA5, GGA8, GGA9, GGA15, GGA17, GGA21 and GGA24 (Table S6). The genomic control inflation factor (λ) calculated for all 17 serum biochemical indicators ranged from 0.98 to 1.01, which indicated mild acceptable false positives except for CREA and ALT (Table S7).

### Loci for the AKP trait

Thirty-one SNPs significantly associated with the AKP trait were identified. A Manhattan plot of the AKP association results is presented in Fig. 5. These 31 SNPs were distributed on GGA3, GGA21 and GGA24, with peaks on GGA21 (5.46–6.67 Mb) and GGA24 (0.27–2.27 Mb). A total of 16 known genes were annotated, including *FAM49A*, *CD30*, *GUCA2A*, *LACTBL1*, *EPHB2*, *C1QA*, *EPHA8*, *PPIH*, *uc_338*, *ROBO3*, *DCPS*, *KIRREL3*, *BARX2B*, *APLP2*, *ZBTB44* and *OPCML*. The most significant SNP markers on GGA24 affecting AKP were rs14292105 and rs14292106, located downstream of the *DCPS* gene (-log_10_(P) = 12.21). Detailed descriptions of the significant SNPs are given in Table S6.

### Loci for the CHE trait

Sixty-eight SNPs were identified to be significantly associated with CHE (Fig. 5 and Table S6); these SNPs were located within a 4.7 Mb (19.0-23.7 Mb) region with a peak on GGA9. Twenty known genes were annotated within the region, including *FNDC3B*, *TNIK*, *EIF5A2*, *PRKCI*, *GPR160*, *LRRC34*, *MECOM*, *BCHE*, *OTOL1*, *PPM1L*, *IQCJ*, *MFSD1*, *RSRC1*, *VEPH1*, *CCNL1*, *SSR3*, *RAP2B*, *ARHGEF26*, *MINDY4B* and *EIF2A*. The most significant SNP marker affecting CHE was located at the intergenic region of the *ENSGALG00000009511* and *ENSGALG00000030029* genes.

### Loci for the GGT trait

A total of 130 SNPs spanning a 6.18 Mb region between 6.51 (rs14020787) and 12.69 (rs314303980) Mb on GGA15 were significantly associated with the GGT trait (Fig. 5 and Table S6). Forty-eight known genes were annotated for these SNPs. Interestingly, GGT2 and GGT5 genes were not included in these 48 genes due to the lack of significant SNPs at these loci. However, we found that both GGT2 and GGT5 genes were within the above mentioned 6.18 Mb region.

### Significant loci for the other five traits

GWAS analyses were also performed for other traits, and significant SNPs were identified for five of them, including AMY, LDH, GLU, HDL and TP. Three SNPs identified at the 14.1 Mb site of GGA8 were found to be associated with AMY; these SNPs were all located within the intergenic region of the *RPL5* and *ENSGALG00000029582* genes. One SNP (rs736356742, -log_10_(*P*) = 6.19) marker that was significantly associated with LDH was located in the intergenic region between the *PTPN5* and *PTPRJ* genes at the 12.8 Mb site of GGA5. One SNP marker (rs315358973, -log_10_(*P*) = 5.91) for GLU was identified on GGA17 downstream of the *TNC* gene. One SNP marker (rs794372306, -log_10_(*P*) = 6.07) for HDL was identified on GGA1 within the intergenic region of the *AQP11* and *PAK1* genes. One SNP (rs734055122, -log_10_(*P*) = 7.55) located within the Coatomer Protein Complex Subunit Gamma 2 (*COPG2*) gene was identified to be associated with TP.

## Discussion

Serum biochemical indicators are blood parameters linked to physiological traits and are often regarded as direct reflections of metabolism and health in animals. To the best of our knowledge, no published study has systematically demonstrated the associations between some or all of the 17 serum biochemical indicators and genomic loci using a GWAS of SNPs in chickens. QTLs related to 8 of the 17 indicators (HDL, AKP, CHE, GGT, CREA, ALT, AST and AMY) in chickens have yet to be reported in the Chicken QTL Database (https://www.animalgenome.org/cgi-bin/QTLdb/GG/index). In our study, we identified significant associations for 8 traits, including AKP, CHE, GGT, AMY, LDH, GLU, HDL and TP. In addition, we performed an analysis of phenotypic correlation to use GWAS find genetic-based associations for some strongly correlated phenotypics. Unfortunately, none of the strongly correlated phenotypic, such as ALB, TP, and GLO, identified significantly associated SNP in the GWAS analysis.

AKP is a family of enzymes exists in almost all animal tissues that catalyse the hydrolysis phosphate esters. AKP in serum mainly originates in the skeleton and liver which often used to diagnose and monitor skeletal or liver disease in the clinic. The levels of AKP in serum is under considerable genetic control according to the Gene variants and heritabilities in swine and human [16, 17]. However, QTL influencing AKP in chicken have not yet been reported. In this study, three QTLs for the AKP trait were identified on GGA3, GGA21 and GGA24, which included sixteen genes suggests that AKP in chicken also under considerable genetic control. Variation within the *ALPL* gene is related to the variation of human serum AKP and bone mineral density [18]. The *ALPL* gene has been proposed as a candidate gene for the AKP trait in pigs and humans [19, 20]. We found that the *ALPL* gene was not directly annotated in our results, but it was located within the QTL on GGA21 which corresponds to a QTL for liver/bone/kidney-derived AKP (ALPL) serum activity. Therefore, a variant or linked variant within the *ALPL* gene may be responsible for the QTL effect on GGA21 in cis-acting substances in this study. In this study, another QTL related to AKP trait were identified on GGA24. The QTL was also identified in the case-control-based GWAS study of the AKP trait mentioned in the background with VVD broilers and sound broilers, the candidate genes located in the QTL were involved in the skeletal development and bone disease. Genetic variation loci of *ST3GAL4* gene located in the QTL were closely related to AKP in VVD broilers in their subsequent study which indicated *ST3GAL4* plays an important role in serum AKP and the skeletal development [3]. Other wise, QTLs associated with AKP activity reported before were mainly involved in growth and bone formation [20, 21]. We postulate that *ST3GAL4* or other genes within the QTL may have a direct or indirect regulatory relationship with the *ALPL* gene to influence the level of AKP in serum and the skeletal development in chickens. Of course, further evidence is needed to understand the regulatory mechanisms. Moreover, with no QTL on GGA9, the location of the intestinal-derived AKP (*ALPI*) gene, the corresponding ALPI enzyme does not seem to be involved in AKP variation in F2 chickens in this study.

CHE is an esterase that can break down choline esters and its activity is related to a family of glycoprotein. There are two types of CHE in vertebrates, Acetylcholinesterase (AChE) and butylcholinesterase (BChE). The former is found in the postsynaptic membrane at the neuromuscular junction, while the latter is synthesized in the liver and has been reported may play a role in lipid metabolism [22]. The activity of CHE in serum is dominated by BChE, the enzyme is often used as an indicator of liver status disease in medical and veterinary trials. Although serum CHE is widely used as a clinical indicator, there are few reports on related QTLs. A previous GWAS on humans has shown that three independent loci on or near the *BCHE* gene are significantly associated with CHE activity in serum [23]. In ducks, Zhu et al [21]. identified 7 SNPs near the *BCHE* gene were significant associations with BChE activity. Consistent with these findings, a QTL for the CHE trait on GGA9 was detected in our study, and the *BCHE* gene within the QTL was also detected to be significantly associated with CHE activity. The above studies of different species all show that CHE activity in serum is significantly associations with *BCHE* gene, which further confirmed that CHE activity in serum is mainly BCHE synthesized in the liver. We further annotated 19 other genes in this QTL on GGA9, while there are few detailed reports on the relationship between CHE and these genes. The most plausible hypothesis is that one or several genes within this QTL might have direct or indirect regulatory effects on *BCHE* gene expression and then affect CHE activity. More molecular studies are needed to reveal the underlying mechanisms of CHE activity.

GGT is a hepatobiliary enzyme synthesized by hepatocytes and intrahepatic bile duct epithelial cells, and its elevated activity is an important predictor of metabolic syndrome. Elevated GGT is a sign of liver disease, and continuously elevated GGT increases the risk of fatty liver disease [24]. Notably, no QTLs for the GGT trait have been reported in any species, thus, our study may be the first to report QTLs that affect GGT level. The QTLs we identified were located within a 6.18 Mb region (6.51 ~ 12.69 Mb) on GGA15, and the *GGT2* and *GGT5* genes were located within this QTL. *GGT2* and *GGT5* is members of the γ-glutamyl transpeptidase gene family which including *GGT1*, *GGT6*, *GGT7* and so on. The QTL for the GGT activity in serum was identified in the genomic region where *GGT2* and *GGT5* genes were located, and it was speculated that the GGT activity in serum was mainly regulated by these two genes.

One significant SNP located at the 12.8 Mb site of GGA5 was identified to be associated with the LDH trait, and the L-lactate dehydrogenase A chain (*LDHA*) that belongs to the lactate dehydrogenase family was annotated to a site 0.2 Mb upstream of the 12.8 Mb site on GGA5. Gene Ontology annotations related to *LDHA* include oxidoreductase activity and L-lactate dehydrogenase activity. Therefore, a variant or linked variant within the *LDHA* gene may be responsible for the QTL effect on GGA5 associated with the LDH trait in this study and the *LDHA* gene could be considered the site of a candidate gene.

The GLU trait was mapped to GGA17 in our study. A previous study has mapped GLU traits elsewhere on GGA17[25]. In addition, a total of 23 QTLs on different chromosomes have been associated with GLU levels in studies based on different experimental designs [13, 25–30]. For example, Hee-Bok Park et al. performed QTL analysis on an F2 population with 874 individuals composing 75 full-sib families and found that one QTL on GGA20 at the 41 Mb site and one QTL on GGA27 at the 21 Mb site were related to the GLU trait [28]. The *TNC* gene was also annotated to a locus we detected; this gene has been demonstrated to be highly associated with a wide range of diseases related to inflammation, including diabetes [31]. In addition, the *TNC* gene is involved in the regulation of adipocyte differentiation and lipid deposition, and the expression of the porcine *TNC* gene is associated with meat quality [32]. Fat is an important source of stored energy, while sugar mainly provides energy for metabolism; however, fat and sugar can be interconverted. The *TNC* gene may affect GLU levels by regulating the relationship between fat deposition and sugar metabolism and thus may be a candidate gene for the GLU trait.

HDL is an antiatherosclerotic plasma lipoprotein that removes cholesterol from atherosclerotic blood vessels and transports it to the liver for metabolic clearance [33]. In our study, the HDL trait was mapped to the region on GGA1 containing the *AQP11* and *PAK1* genes. To our knowledge, no QTLs on GGA1 that are associated with HDL levels in chickens have been reported thus far; however, the interval in which the *AQP11* and *PAK1* genes are located in the pig genome is also associated with HDL levels [34]. *PAK1* plays important roles in a variety of cellular processes, including cell polarity, motility, survival and proliferation [35]. The increase in PAK1 phosphorylation in human atherosclerotic arteries suggests a role for this gene in human atherogenesis [35]. The *PAK1* gene also promotes activation of inflammatory pathways in endothelial cells by fluid shear stress, which is the initial stimulator of atherosclerosis [36]. Transport of cholesterol via HDL from peripheral tissues to the liver is a mechanism associated with very advanced antiatherosclerosis [33]. *PAK1* mediates the downregulation of scavenger receptor B1 promoter activity to play a role in cholesterol transport [37]. Notably, *PAK1* deficiency leads to enhancement of cholesterol efflux and upregulation of reverse cholesterol transporters, which suggests that *PAK1* exerts negative modulatory effects on transporters and may promote lipid retention in inflamed arteries, causing atherogenesis [35]. Therefore, *PAK1* may be a candidate gene affecting serum levels of HDL.

The serum AMY trait was mapped to the 14.1 Mb site of GGA8, 3.1 Mb downstream of the *AMY1A* gene. The *AMY1A* gene encodes AMY, a secreted protein that hydrolyzes 1,4-alpha-glucoside bonds in oligosaccharides and polysaccharides and thus catalyzes the first step in the digestion of dietary starch and glycogen. Genetic variation in this genomic interval may affect the expression of *AMY* genes or the translation of proteins through indirect effects, thereby regulating the levels of AMY in serum.

TP is an indicator of growth and nutritional status [38]. The level of serum TP of fat pigs are higher than that of lean pigs [9], and the level of serum TP has been proved to be the best indicator for early fat estimation in pigs [10]. In addition, TP can also be used as a potential biomarker to select the fat traits of ducks [21]. Previous research has identified six QTLs related to the TP trait that are located on GGA1, GGA2, GGA12, GGA18 and GGAZ [25]; however, our study has identified a new QTL on GGA1 that is located within the *COPG2* gene. The coatomer is a cytosolic protein complex that further mediates biosynthetic protein transport from the endoplasmic reticulum (ER) via the Golgi to the trans-Golgi network. The *COPG2* gene was enriched for the Delta508-CFTR traffic/ER-to-Golgi in cystic fibrosis (CF) pathway, vesicle-mediated transport pathway, and protein metabolism pathway, among other pathways (https://www.genecards.org/cgi-bin/carddisp.pl?gene=COPG2&keywords=COPG2). Thus, *COPG2* may regulate the TP trait by affecting protein transport and metabolism.

In summary, although the QTLs and candidate genes identified by our GWAS have not been reported in other studies, we have confirmed the associations of the QTLs/candidate genes with serum biochemical indicator traits in other species or the biological functions of the potential candidate genes. The different association results among different studies might have been caused by the different experimental populations, different genetic backgrounds and different stages at which the traits were measured.

## Conclusion

This study identified 10 novel QTL regions that affected 8 serum biochemical indicators based on a Gushi×Anka F2 resource population using genotyping by sequencing. We propose that the *ALPL*, *BCHE*, and *GGT2*/*GGT5* genes might affect the AKP, CHE and GGT traits, respectively. The results provide a basis for uncovering the molecular mechanism of serum biochemical indicator regulation in chicken.

## Methods

### Establishment of the experimental population

The F_2_ resource population used in this study was created by the Henan Innovative Engineering Research Center of the Poultry Germplasm Resource as described previously [39]. The F_2_ resource population was established with a slow-growing breed (the Gushi chicken) and a fast-growing breed (the Anka chicken), which consisted of 4 cross-bred families (Anka cocks mated with Gushi hens) and 3 reciprocal families (Gushi cocks mated with Anka hens) as described previously [39]. A total of 860 chickens were obtained in the F_2_ population. All chickens were kept in cages under the same environmental conditions with free access to feed and water according to the National Research Council recommendations (1994).

### Phenotypic measurements

Blood samples were collected for all 860 F_2_ chickens after the chickens were slaughtered at 12 weeks of age. Then, serum samples were separated by a standard protocol and stored at -80 °C for measurement of serum biochemical indicators. A total of 17 serum biochemical indicators, including CHO, TGs, HDL, LDL, glucose (GLU), AKP, CHE, creatine phosphokinase (CK), γ-glutamyl transpeptidase (GGT), lactate dehydrogenase (LDH), CREA, TP, GLO, ALB, ALT, AST, and amylase (AMY), were measured with kits from Nanjing Jiancheng (Nanjing, China). The measurement methods have been previously detailed by Han et al.[40, 41] All phenotypic data were analyzed with normality tests. If a phenotype did not follow the normal distribution (asymptotic *P* < 0.05), it would be ranked as normal scores with the Tukey method, and then transformed data were used in the following genetic analyses. The pheatmap R package (1.0.12) was applied to visualize the Pearson correlations between phenotypes.

### Genotyping and quality control

Genomic DNA was extracted from blood samples with a DNeasy Blood and Tissue Kit according to the manufacturer’s instructions (Qiagen, Hilden, Germany). All DNA samples were diluted to 40 ng/µl, and 200 ng of DNA was digested with a combination of EcoRI and MseI for double-digest genotyping by sequencing (ddGBS)[42]. Eight libraries were constructed, and a total of 768 samples were genotyped. Sequencing experiments were performed via paired-end 2 × 150 nt runs on an Illumina HiSeq X Ten platform controlled by data collection software. The image data were exported, transformed into raw data and stored in FASTQ (fq) format after sequencing. The 150 bp paired-end Illumina reads containing the adapter sequence were deleted, and those containing more than 50% low-quality bases or more than 5% N bases were removed. A quality control report for the filtered reads was generated by FastQC software. All downstream analyses were based on the resulting clean data.

The TASSEL-GBS analysis pipeline (version 5.2.31)[43, 44] was used to call single-nucleotide polymorphisms (SNPs), and the reads were aligned to the chicken reference genome Gallus_gallus-5.0 (*Gallus gallus* 5.0) using Bowtie2 (version 2.3.0) [45]. Then, we used VCFtools (version 0.1.13) [46] to filter the raw SNPs with the following parameters: a minor allele frequency (MAF) greater than 5% (maf 0.05), a genotype quality greater than 98 (minGQ 98), a genotype depth greater than 5 (minDP 5), retention of only biallelic markers (max-alleles 2; min-alleles 2), conformation to Hardy-Weinberg equilibrium (hwe 0.0001) and a maximum missing rate less than 0.4. Individual samples were excluded with two-sided call rates of less than 0.3. We merged the SNPs of paired-end reads and imputed the ungenotyped markers according to the information on the remaining SNPs with Beagle4.0 software [47]. SNPs located on sex chromosomes (GGAZ and GGAW) were removed before the GWAS was performed. We have analyzed the population structure and removed the abnormal individuals in previous study [48].

### Single-trait GWAS

The top two principal components (PCs) were used as covariates in the mixed model [48]. A genomic relationship matrix was constructed with the SNPs using GCTA software and used as a random effect in the mixed model.

A GWAS was carried out on serum biochemical indicators using the following mixed linear model (MLM) equation in the GCTA program:

$$y{\rm{ }} = {\rm{ }}X\beta {\rm{ }} + {\rm{ }}{X_{SNP}}{\beta _{SNP}} + {\rm{ }}Zu{\rm{ }} + {\rm{ }}e$$

where y is the vector of individual observations, β is the vector of fixed environmental effects (sex, batch and top two PCs), u is the vector of random direct additive genetic effects obtained from MVN (u ~ 0, σ^2u), and e is the vector of random residual errors obtained from MVN (u ~ 0, σ^2e). The X and Z are design matrices that relate individuals’ records to their fixed effects (β) and additive genetic effects (u), respectively. X_SNP_ is the incidence matrix for the SNP markers and β_SNP_ is the regression coefficient for each SNP (SNP effects).

The genome-wide significance (*P* value threshold) was adjusted with the Bonferroni method based on the number of independent tests. We calculated the number of genome-wide independent markers using the PLINK [49] --indep-pairwise command with a window size of 25 SNPs, a step of 5 SNPs, and an r^2^ threshold of 0.1. The significance level was set as 1.90E-06 (0.05/26,352; -log_10_(P) > 5.72) based on the 26,352 independent markers. Manhattan and quantile-quantile (Q-Q) plots were obtained using the CMplot package (https://github.com/YinLiLin/R-CMplot) within R software (version 3.6.1) (http://www.r-project.org/).

### Post-GWAS analysis

The Ensembl genome database and the SNPEff (version 4.1) program [50] were used to obtain information about SNPs and relevant gene annotations for the GWAS results. Additionally, the plotrix (3.7-8), latticeExtra (0.6–29), scales (1.1.0) and ggplot2 (3.3.0) packages within R software (version 4.0.0) were applied to visualize the distributions of chromosome length and SNPs in the chicken genome. The CMplot package was used to visualize the density of SNPs in the chicken genome.

## Electronic supplementary material

Below is the link to the electronic supplementary material.

**Additional file 1. Table S1.** Descriptive statistics for the serum biochemical indicators

**Additional file 2. Table S2**. Pearson correlation coefficients between serum biochemical indicators

**Additional file 3. Table S3.** Statistics for the sequenced data

**Additional file 4. Table S4.** Distribution of SNPs discovered from 734 individuals across chromosomes

**Additional file 5. Table S5.** Annotated results of SNPs from GBS sequencing

**Additional file 6. Table S6.** Significant SNPs for serum biochemical indicators.

**Additional file 7. Table S7.** The genomic inflation factor (λ statistic) for serum biochemical indicators

**Additional file 8. Figure S1**

## Data Availability

The authors declare that the data supporting the findings of this study are available within the article and its supplementary information files. All the raw sequences have been deposited in the NCBI database Sequence Read Archive with the accession numbers SRR12532401–SRR12532408 (BioProject number PRJNA659316).
