# Supplementary material for: Genome-wide association study of 17 serum biochemical indicators in a chicken F2 resource population
Source: BMC Genomics. 2023 Mar 2;24:98. doi: 10.1186/s12864-023-09206-7 (PMC9983160; doi:10.1186/s12864-023-09206-7)
Supplement: Supplementary file 1 — Additional file 1. Table S1. Descriptive statistics for the serum biochemical indicators [file 12864_2023_9206_MOESM1_ESM.docx]

**Table S1. Descriptive statistics for the serum biochemical indicators.**

| **serum biochemical indicators** | **Units** | **No. of records^1^** | **Min** | **Max** | **Mean** | **SD** | **Coefficient of variation (%)** | **Kolmogorov-Smirnov Z^2^** | **asymptotic-P^3^** |
| --- | --- | --- | --- | --- | --- | --- | --- | --- | --- |
|  |  |  |  |  |  |  |  |  |  |
| CHO | mmol/L | 664 | 1.29 | 5.51 | 3.14 | 0.72 | 22.87 | 0.86 | 0.46 |
| TG | mmol/L | 662 | 0.06 | 0.83 | 0.41 | 0.10 | 23.98 | 1.54 | 0.02 |
| HDL | mmol/L | 663 | 0.11 | 3.05 | 1.98 | 0.41 | 20.51 | 0.83 | 0.50 |
| LDL | mmol/L | 661 | 0.08 | 3.09 | 1.01 | 0.41 | 40.46 | 1.36 | 0.05 |
| GLU | mmol/L | 651 | 0.02 | 16.76 | 8.69 | 3.49 | 40.18 | 1.32 | 0.06 |
| AKP | U/L | 652 | 122.00 | 2660.00 | 706.34 | 489.61 | 69.32 | 3.97 | 0.00 |
| CHE | KU/L | 660 | 0.10 | 3.50 | 1.89 | 0.50 | 26.31 | 2.01 | 0.00 |
| CK | IU/L | 655 | 1304.00 | 12379.00 | 7239.36 | 1870.89 | 25.84 | 1.70 | 0.01 |
| GGT | U/L | 641 | 1.00 | 33.00 | 15.57 | 5.56 | 35.73 | 1.95 | 0.00 |
| LDH | U/L | 661 | 922.00 | 4423.00 | 2771.61 | 478.37 | 17.26 | 0.62 | 0.84 |
| CREA | umol/L | 294 | 0.00 | 34.00 | 3.83 | 4.98 | 129.93 | 3.79 | 0.00 |
| TP | g/L | 661 | 24.00 | 72.90 | 42.87 | 7.76 | 18.11 | 2.29 | 0.00 |
| GLO | g/L | 664 | 12.70 | 52.80 | 26.35 | 6.55 | 24.88 | 2.86 | 0.00 |
| ALB | g/L | 662 | 10.80 | 29.50 | 16.64 | 2.19 | 13.17 | 2.12 | 0.00 |
| ALT | U/L | 504 | 0.00 | 21.00 | 1.77 | 1.78 | 100.49 | 4.52 | 0.00 |
| AST | U/L | 660 | 152.00 | 687.00 | 285.19 | 67.10 | 23.53 | 3.03 | 0.00 |
| AMY | U/L | 628 | 45.00 | 1357.00 | 430.28 | 191.81 | 44.58 | 2.30 | 0.00 |

^1^Number of individuals.

^2^Z value from the normality test (SPSS nonparametric 1-sample Kolmogorov-Smirnov test).

^3^Significance value from the normality test (SPSS nonparametric 1-sample Kolmogorov-Smirnov test).

Note：
