# Supplementary material for: Genome-wide association study of 17 serum biochemical indicators in a chicken F2 resource population
Source: BMC Genomics. 2023 Mar 2;24:98. doi: 10.1186/s12864-023-09206-7 (PMC9983160; doi:10.1186/s12864-023-09206-7)
Supplement: Supplementary file 2 — Additional file 2. Table S2. Pearson correlation coefficients between serum biochemical indicators [file 12864_2023_9206_MOESM2_ESM.docx]

**Table S2. Pearson correlation coefficients between serum biochemical indicators.**

| **Traits** | **CHO** | **TG** | **HDL** | **LDL** | **GLU** | **AKP** | **CHE** | **CK** | **GGT** | **LDH** | **CREA** | **TP** | **GLO** | **ALB** | **ALT** | **AST** | **AMY** |
| --- | --- | --- | --- | --- | --- | --- | --- | --- | --- | --- | --- | --- | --- | --- | --- | --- | --- |
| CHO | 1 | .075 | .760^**^ | .760^**^ | .226^**^ | -.024 | .262^**^ | .002 | .251^**^ | -.019 | -.072 | .193^**^ | .147^**^ | .199^**^ | .011 | .253^**^ | -.002 |
| TG | .075 | 1 | -.012 | .108^**^ | -.258^**^ | .120^**^ | .113^**^ | .137^**^ | -.074 | .256^**^ | -.181^**^ | .223^**^ | .239^**^ | .138^**^ | -.134^**^ | -.017 | .061 |
| HDL | .760^**^ | 0 | 1 | .379^**^ | .247^**^ | -.002 | .285^**^ | .002 | .223^**^ | -.004 | -.009 | .044 | -.031 | .167^**^ | -.026 | .228^**^ | .028 |
| LDL | .760^**^ | .108^**^ | .379^**^ | 1 | .112^**^ | -.003 | .235^**^ | .075 | .135^**^ | .042 | .037 | .300^**^ | .290^**^ | .191^**^ | .037 | .155^**^ | -.017 |
| GLU | .226^**^ | -.258^**^ | .247^**^ | .112^**^ | 1 | .068 | .016 | -.095^*^ | .128^**^ | -.308^**^ | -.040 | -.296^**^ | -.324^**^ | -.130^**^ | .026 | -.061 | .031 |
| AKP | -.024 | .120^**^ | -.002 | -.003 | .068 | 1 | -.048 | .002 | -.002 | -.063 | -.055 | -.008 | .025 | -.041 | -.037 | -.058 | .100^*^ |
| CHE | .262^**^ | .113^**^ | .285^**^ | .235^**^ | .016 | -.048 | 1 | .132^**^ | .146^**^ | .137^**^ | -.042 | .193^**^ | .133^**^ | .207^**^ | .009 | .148^**^ | -.014 |
| CK | .002 | .137^**^ | .002 | .075 | -.095^*^ | .002 | .132^**^ | 1 | -.092^*^ | .345^**^ | -.023 | .095^*^ | .078^*^ | .099^*^ | -.050 | .231^**^ | .008 |
| GGT | .251^**^ | -.074 | .223^**^ | .135^**^ | .128^**^ | -.002 | .146^**^ | -.092^*^ | 1 | -.048 | -.054 | .050 | .046 | .000 | .014 | .090^*^ | -.033 |
| LDH | -.019 | .256^**^ | -.004 | .042 | -.308^**^ | -.063 | .137^**^ | .345^**^ | -.048 | 1 | -.008 | .393^**^ | .376^**^ | .268^**^ | -.015 | .161^**^ | .104^**^ |
| CREA | -.072 | -.181^**^ | -.009 | .037 | -.040 | -.055 | -.042 | -.023 | -.054 | -.008 | 1 | -.002 | -.016 | .049 | -.078 | -.052 | -.020 |
| TP | .193^**^ | .223^**^ | .044 | .300^**^ | -.296^**^ | -.008 | .193^**^ | .095^*^ | .050 | .393^**^ | -.002 | 1 | .957^**^ | .675^**^ | .085 | .078^*^ | .070 |
| GLO | .147^**^ | .239^**^ | -.031 | .290^**^ | -.324^**^ | .025 | .133^**^ | .078^*^ | .046 | .376^**^ | -.016 | .957^**^ | 1 | .490^**^ | .062 | .038 | .078 |
| ALB | .199^**^ | .138^**^ | .167^**^ | .191^**^ | -.130^**^ | -.041 | .207^**^ | .099^*^ | .000 | .268^**^ | .049 | .675^**^ | .490^**^ | 1 | .120^**^ | .159^**^ | .013 |
| ALT | .011 | -.134^**^ | -.026 | .037 | .026 | -.037 | .009 | -.050 | .014 | -.015 | -.078 | .085 | .062 | .120^**^ | 1 | -.047 | .070 |
| AST | .253^**^ | -.017 | .228^**^ | .155^**^ | -.061 | -.058 | .148^**^ | .231^**^ | .090^*^ | .161^**^ | -.052 | .078^*^ | .038 | .159^**^ | -.047 | 1 | -.003 |
| AMY | -.002 | .061 | .028 | -.017 | .031 | .100^*^ | -.014 | .008 | -.033 | .104^**^ | -.020 | .070 | .078 | .013 | .070 | -.003 | 1 |

The numbers are the Pearson correlation coefficients of pairs of traits, the positive numbers represent positive correlations, and the negative numbers represent negative correlations. **, significant correlation at the 0.01 level (two-sided); *, significant correlation at the 0.05 level (two-sided).
