# Supplementary material for: Genome-wide association study of 17 serum biochemical indicators in a chicken F2 resource population
Source: BMC Genomics. 2023 Mar 2;24:98. doi: 10.1186/s12864-023-09206-7 (PMC9983160; doi:10.1186/s12864-023-09206-7)
Supplement: Supplementary file 3 — Additional file 3. Table S3. Statistics for the sequenced data [file 12864_2023_9206_MOESM3_ESM.docx]

**Table S3. Statistics for the sequenced data.**

| **Lane^1^** | **Bases (G)^2^** | **Total Reads^3^** | **Q30**  **(%)** | **Q20**  **(%)** | **R1 Reads^4^** | **R1 Good Barcode Reads^5^** | **R1 good ratio^6^** | **R2 Reads^4^** | **R2 Good Barcode Reads^5^** | **R2 good ratio^6^** | **Filter^7^** |
| --- | --- | --- | --- | --- | --- | --- | --- | --- | --- | --- | --- |
|  |  |  |  |  |  |  |  |  |  |  |  |
| L1 | 121 | 808,639,426 | 92 | 96 | 404,319,713 | 295,785,461 | 0.73 | 404,319,713 | 351,873,955 | 0.87 | 7 |
| L2 | 125 | 832,928,728 | 93 | 97 | 416,464,364 | 295,646,345 | 0.71 | 416,464,364 | 357,060,282 | 0.86 | 5 |
| L3 | 127 | 848,358,674 | 93 | 97 | 424,179,337 | 299,066,848 | 0.71 | 424,179,337 | 366,820,501 | 0.86 | 7 |
| L4 | 144 | 961,606,222 | 93 | 97 | 480,803,111 | 404,804,456 | 0.84 | 480,803,111 | 413,407,380 | 0.86 | 3 |
| L5 | 145 | 968,786,086 | 93 | 97 | 484,393,043 | 405,210,921 | 0.84 | 484,393,043 | 418,048,747 | 0.86 | 3 |
| L6 | 142 | 948,387,596 | 92 | 97 | 474,193,798 | 402,730,494 | 0.85 | 474,193,798 | 418,114,080 | 0.88 | 3 |
| L7 | 141 | 940,898,906 | 92 | 97 | 470,449,453 | 409,728,378 | 0.87 | 470,449,453 | 415,260,489 | 0.88 | 3 |
| L8 | 142 | 948,527,654 | 92 | 97 | 474,263,827 | 403,069,787 | 0.85 | 474,263,827 | 414,036,702 | 0.87 | 3 |
| Total | 1,089 | 7,258,133,292 | 92.63 | 96.57 | 3,629,066,646 | 2,916,042,690 | 0.80 | 3,629,066,646 | 3,154,622,136 | 0.87 | 34 |

^1^Lanes 1-8 represent the 8 libraries. Eight libraries of 96 samples each were constructed, and a total of 768 samples were subjected to genotyping.

^2^“Bases” refers to the amount of sequencing data.

^3^“Total Reads” is the sum of the R1 Reads and R2 Reads.

^4^“R1 Reads” and “R2 Reads” refer to the reads from paired-end sequencing (PE150).

^5^A good barcode read is a sequence read that matches one of the barcodes used in ddGBS. “R1 Good Barcode Reads” and “R2 Good Barcode Reads” refer to the good barcode reads for the paired-end sequences (PE150).

^6^R1 good ratio = R1 Good Barcode Reads/R1 Reads. R2 good ratio = R2 Good Barcode Reads/R2 Reads.

^7^“Filter” refers to the number of filtered individuals.
