# Supplementary material for: Genome-wide association study of 17 serum biochemical indicators in a chicken F2 resource population
Source: BMC Genomics. 2023 Mar 2;24:98. doi: 10.1186/s12864-023-09206-7 (PMC9983160; doi:10.1186/s12864-023-09206-7)
Supplement: Supplementary file 4 — Additional file 4. Table S4. Distribution of SNPs discovered from 734 individuals across chromosomes [file 12864_2023_9206_MOESM4_ESM.docx]

**Table S7. The genomic inflation factor (λ statistic) for serum biochemical indicators.**

| **Serum biochemical indicators** | **λ(sex, batch)** |
| --- | --- |
| CHO | 1.01 |
| TG | 1.00 |
| HDL | 1.00 |
| LDL | 1.01 |
| GLU | 0.99 |
| AKP | 0.98 |
| CHE | 0.98 |
| CK | 1.00 |
| GGT | 0.99 |
| LDH | 1.00 |
| TP | 1.00 |
| GLO | 1.00 |
| ALB | 1.01 |
| AST | 0.99 |
| AMY | 1.00 |
| Average | 1.00 |
