# Supplementary material for: Genome-wide association study of 17 serum biochemical indicators in a chicken F2 resource population
Source: BMC Genomics. 2023 Mar 2;24:98. doi: 10.1186/s12864-023-09206-7 (PMC9983160; doi:10.1186/s12864-023-09206-7)
Supplement: Supplementary file 5 — Additional file 5. Table S5. Annotated results of SNPs from GBS sequencing [file 12864_2023_9206_MOESM5_ESM.docx]

**Table S5. Annotated results of SNPs from GBS sequencing.**

| **Type^1^** | **Count^2^** | **Percent (%)^3^** |
| --- | --- | --- |
| Exon | 7171 | 1.09 |
| Intergenic | 177428 | 26.89 |
| Intron | 274893 | 41.66 |
| Downstream | 97260 | 14.74 |
| Upstream | 99200 | 15.03 |
| UTR_3_Prime | 2494 | 0.38 |
| UTR_5_Prime | 469 | 0.07 |
| Other regions | 955 | 0.15 |

^1^Genomic region of SNPs.

^2^Number of SNPs by region.

^3^Ratio of SNPs by region.
