# Supplementary material for: Genome-wide association study of 17 serum biochemical indicators in a chicken F2 resource population
Source: BMC Genomics. 2023 Mar 2;24:98. doi: 10.1186/s12864-023-09206-7 (PMC9983160; doi:10.1186/s12864-023-09206-7)
Supplement: Supplementary file 6 — Additional file 6. Table S6. Significant SNPs for serum biochemical indicators. [file 12864_2023_9206_MOESM6_ESM.docx]

**Table S4. Distribution of SNPs discovered from 734 individuals across chromosomes.**

| **Chromosome^1^** | | **Genes^2^** | **Variants (SNPs)^3^** | **Density (SNPs/Mb)^4^** | **Variants rate(bp/SNP)^5^** | **Novel^6^** |
| --- | --- | --- | --- | --- | --- | --- |
| **Chr.** | **Size (bp)** |  |  |  |  |  |
| Chr1 | 196,202,544 | 2,962 | 67,301 | 343 | 2,915 | 6,773 |
| Chr2 | 149,560,735 | 1,945 | 52,480 | 351 | 2,849 | 5,612 |
| Chr3 | 111,302,122 | 1,715 | 38,172 | 343 | 2,915 | 3,682 |
| Chr4 | 91,282,656 | 1,547 | 32,011 | 351 | 2,851 | 3,129 |
| Chr5 | 59,825,302 | 1,295 | 21,434 | 358 | 2,791 | 1,945 |
| Chr6 | 35,467,016 | 724 | 13,395 | 378 | 2,647 | 1,202 |
| Chr7 | 36,946,936 | 699 | 12,813 | 347 | 2,883 | 1,360 |
| Chr8 | 29,963,013 | 709 | 9,749 | 325 | 3,073 | 953 |
| Chr9 | 24,091,566 | 615 | 8,641 | 359 | 2,788 | 823 |
| Chr10 | 20,435,342 | 583 | 7,155 | 350 | 2,856 | 831 |
| Chr11 | 20,218,793 | 484 | 6,613 | 327 | 3,057 | 633 |
| Chr12 | 19,948,154 | 482 | 7,798 | 391 | 2,558 | 930 |
| Chr13 | 18,407,460 | 498 | 6,329 | 344 | 2,908 | 585 |
| Chr14 | 15,595,052 | 537 | 5,291 | 339 | 2,947 | 413 |
| Chr15 | 12,762,846 | 462 | 4,316 | 338 | 2,957 | 534 |
| Chr16 | 652,338 | 115 | 173 | 265 | 3,770 | 71 |
| Chr17 | 10,956,400 | 390 | 3,389 | 309 | 3,232 | 307 |
| Chr18 | 11,053,727 | 405 | 3,613 | 327 | 3,059 | 368 |
| Chr19 | 9,979,828 | 410 | 2,936 | 294 | 3,399 | 214 |
| Chr20 | 14,109,371 | 472 | 4,922 | 349 | 2,866 | 547 |
| Chr21 | 6,862,722 | 304 | 2,339 | 341 | 2,934 | 234 |
| Chr22 | 4,729,743 | 174 | 804 | 170 | 5,882 | 90 |
| Chr23 | 5,786,528 | 284 | 1,882 | 325 | 3,074 | 121 |
| Chr24 | 6,280,547 | 226 | 2,272 | 362 | 2,764 | 154 |
| Chr25 | 2,906,300 | 312 | 542 | 186 | 5,362 | 85 |
| Chr26 | 5,313,770 | 326 | 1,393 | 262 | 3,814 | 174 |
| Chr27 | 5,655,794 | 430 | 1,577 | 279 | 3,586 | 301 |
| Chr28 | 4,974,273 | 371 | 1,293 | 260 | 3,847 | 200 |
| Chr30 | 24,927 | 6 | 5 | 201 | 4,985 | 4 |
| Chr31 | 49,161 | 5 | 40 | 814 | 1,229 | 40 |
| Chr32 | 78,254 | 17 | 1 | 13 | 78,254 | 1 |
| Chr33 | 1,648,031 | 141 | 315 | 191 | 5,231 | 103 |
| LGE64^7^ | 897,576 | 66 | 320 | 357 | 2,804 | 153 |
| ChrW | 5,160,035 | 50 | 367 | 71 | 14,060 | 366 |
| ChrZ | 82,310,166 | 1,137 | 15,201 | 185 | 5,414 | 2,739 |
| Mean |  |  |  | 309 | 5,787 |  |
| Total | 1,021,439,028 | 20,898 | 336,882 |  |  | 35,677 |
| GWAS_SNPs (Total SNPs except ChrZ and ChrW) | |  | 321314 |  |  |  |

^1^Numbers and sizes of chromosomes in *Gallus gallus* 5.0.

^2^Numbers of genes on chromosomes in *Gallus gallus* 5.0.

^3^Numbers of SNPs on chromosomes as determined by genotyping by sequencing (ddGBS).

^4^Density (SNPs/Mb) = no. of variants/size.

^5^Variant rate (bp/SNP) = size/no. of variants.

^6^SNPs first identified by BLAST analysis with the NCBI chicken dbSNP.

^7^LGE64 is the largest linkage group in the chicken reference genome *Gallus gallus* 5.0.
