# Supplementary figures and images for: Genome-wide association study of 17 serum biochemical indicators in a chicken F2 resource population
Source: BMC Genomics. 2023 Mar 2;24:98. doi: 10.1186/s12864-023-09206-7 (PMC9983160; doi:10.1186/s12864-023-09206-7)

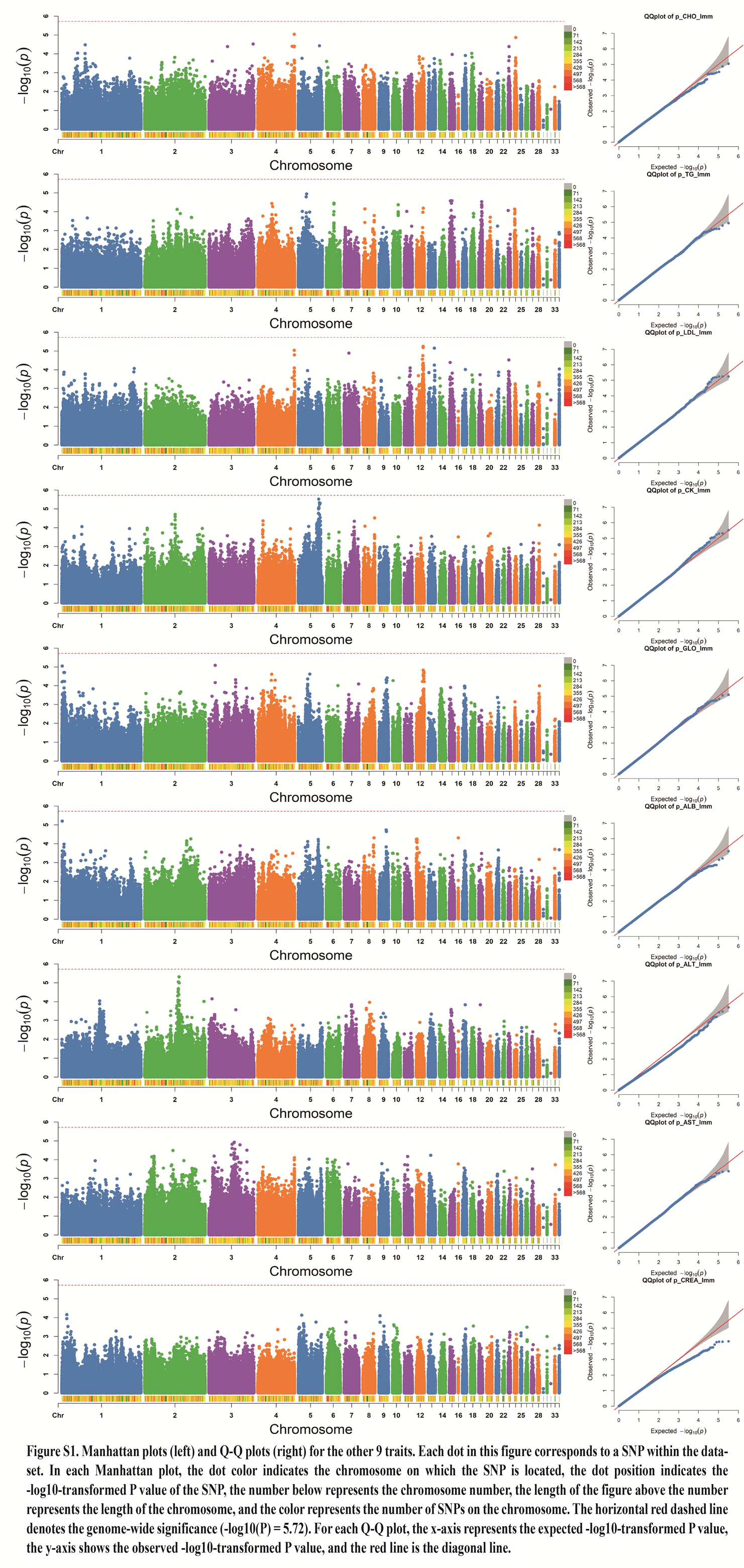

Supplement: Supplementary file 7 — Additional file 7. Table S7. The genomic inflation factor (λ statistic) for serum biochemical indicators [file 12864_2023_9206_MOESM7_ESM.tiff]
